# Comment on human cytomegalovirus seropositivity is associated with reduced patient survival during sepsis

**DOI:** 10.1186/s13054-023-04730-0

**Published:** 2023-11-17

**Authors:** Hongyu Wang, Lin Zhong

**Affiliations:** 1 The Fifth Clinical Medical College of Henan University of Chinese Medicine, Zhengzhou, China; 2 The First People’s Hospital of Pinghu, Pinghu, China

Dear Editor

We read the article by Unterberg et al. published in Critical Care with great interest [1]. The authors found that several biomarkers, such as procalcitonin, IL-10, and IL-6, could predict outcomes only in HCMV-seropositive patients. However, we wish to draw attention to some key aspects that might have been overlooked in the study.

First, the proportion of HCMV reactivation patients found in this study is much lower compared to previous studies [2–5]. The previous studies in this field included immunocompetent patients, whereas this study incorporated a significant number of immunocompromised patients. Why, in this case, does this research show a lower rate of HCMV reactivation compared to what has been reported in previous studies? We inquire whether some patients in this study received CMV prophylactic treatment, as is routine in posttransplant cases, which may reduce the reactivation of CMV. Meanwhile, drug treatments such as rituximab and bortezomib may deplete B cells, thereby reducing the significance of HCMV seropositivity.

Second, the absence of HCMV reactivation in early deceased patients raises the necessity of factoring in early mortality as a competing variable when calculating the incidence of HCMV reactivation. Third, existing research has demonstrated that secondary infections might significantly impact the mortality of HCMV patients [2]. Consequently, incorporating data on secondary infections would yield more accurate and comprehensive insights. The better predictive power of IL-6 and PCT in patients at high risk of secondary infection might be one of the reasons. Finally, simultaneous reactivation of various herpesviruses has been identified in various studies [4, 5]. Thus, we recommend further investigations into the concurrent reactivation of diverse herpesviruses for future research.

In conclusion, while this study holds significant implications for the investigation of biomarkers in HCMV-seropositive patients, there is a need for further refinement, including preventive treatment measures, and the impact of other concurrent herpesviruses. These additional studies will contribute to a more comprehensive understanding of the clinical implications related to HCMV reactivation.

## Data Availability

None.
